# The occurrence of intracranial rhabdoid tumours in mice depends on temporal control of *Smarcb1* inactivation

**DOI:** 10.1038/ncomms10421

**Published:** 2016-01-28

**Authors:** Zhi-Yan Han, Wilfrid Richer, Paul Fréneaux, Céline Chauvin, Carlo Lucchesi, Delphine Guillemot, Camille Grison, Delphine Lequin, Gaelle Pierron, Julien Masliah-Planchon, André Nicolas, Dominique Ranchère-Vince, Pascale Varlet, Stéphanie Puget, Isabelle Janoueix-Lerosey, Olivier Ayrault, Didier Surdez, Olivier Delattre, Franck Bourdeaut

**Affiliations:** 1 Institut Curie, Paris Sciences et Lettres Research University, InsermU830, Laboratoire de Genetique et Biologie des Cancers, 26 rue d'Ulm, 75005 Paris, France; 2 SiRIC- Institut Curie, Laboratoire de Recherche Translationnelle en Oncologie Pédiatrique, 26 rue d'Ulm, 75005 Paris, France; 3 Département de Biologie des Tumeurs, Institut Curie, Service d'anatomie pathologique, 26 rue d'Ulm, 75005 Paris, France; 4 Institut Bergonie, Institut Curie, Unité de génétique somatique, Département de Biologie des Tumeurs, 26 rue d'Ulm, 75005 Paris, France; 5 Institut Curie, Plateforme de pathologie expérimentale, Département de Biologie des Tumeurs, 26 rue d'Ulm, 75005 Paris, France; 6 Centre Léon Bérard, Departement de Biopathologie, 28 Promenade Léa et Napoléon Bullukian, 69008 Lyon, France; 7 Departement de neuropathology, Hopital Sainte-Anne, 1 rue Cabanis, 75014 Paris, France; 8 Université Paris Descartes, 75006 Paris, France; 9 Service de neurochirurgie pédiatrique, Hopital Necker, 149 rue de Sèvres, 75015 Paris, France; 10 Institut Curie, Paris Sciences et Lettres University Research, CNRS UMR 3306, INSERM U1005, Centre Universitaire d'Orsay, 91898 Orsay, France; 11 Département d'oncologie pédiatrique, Institut Curie, 26 rue d'Ulm, 75005 Paris, France

## Abstract

Rhabdoid tumours (RTs) are highly aggressive tumours of infancy, frequently localized in the central nervous system (CNS) where they are termed atypical teratoid/rhabdoid tumours (AT/RTs) and characterized by bi-allelic inactivation of the *SMARCB1* tumour suppressor gene. In this study, by temporal control of tamoxifen injection in *Smarcb1*^flox/flox^;Rosa26-Cre^ERT2^ mice, we explore the phenotypes associated with *Smarcb1* inactivation at different developmental stages. Injection before E6, at birth or at 2 months of age recapitulates previously described phenotypes including embryonic lethality, hepatic toxicity or development of T-cell lymphomas, respectively. Injection between E6 and E10 leads to high penetrance tumours, mainly intra-cranial, with short delays (median: 3 months). These tumours demonstrate anatomical, morphological and gene expression profiles consistent with those of human AT/RTs. Moreover, intra- and inter-species comparisons of tumours reveal that human and mouse RTs can be split into different entities that may underline the variety of RT cells of origin.

## Abstract (web summary)

*SMARCB1* inactivation is prevalent in human atypical teratoid/rhabdoid tumours but a mouse model that accurately phenocopies the human disease is lacking. Here, the authors show that inactivation of *SMARCB1* between E6 and E10 in mice results in tumours that better recapitulate the human phenotype, compared to previously reported models.

## Graphical abstract

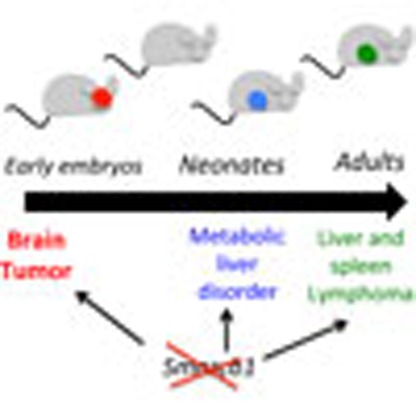

Rhabdoid tumours (RTs) are highly aggressive cancers affecting infants and young children. They occur at various anatomic locations, including kidney, soft parts and the central nervous system (CNS), where they constitute a specific entity termed *atypical teratoid* rhabdoid tumours (AT/RTs)^1^. Expression profilings suggest stem cells as potential cells of origin for RTs, in keeping with their remarkably early onset^2^,^3^,^4^. The main genetic hallmark of all RTs is their unique genomic stability, and *SMARCB1* inactivation is the only recurrent genetic event^5^,^6^. From a clinical point of view, highly intensive treatments are currently evaluated to achieve better survival^7^, but the poor prognosis and the long-term sequels of such intensive treatments in infants remain major causes of concern. Hence, faithful preclinical models are warranted.

Attempts to recapitulate RT in murine models have met with variable success. Although homozygous knockout *Smarcb1* leads to early embryonic lethality, *Smarcb1* heterozygous mice are prone to developing tumours resembling human RTs^8^,^9^,^10^. However, these tumours almost exclusively develop from soft parts of neck with a long latency and a weak penetrance; almost no brain tumour is reported in those models. Conditional inactivation using a *Smarcb1*^inv^;Mx-Cre model leads to highly aggressive, fully penetrant tumours, which were demonstrated to be CD8(+), mature peripheral T-cell lymphomas^11^. Thus a genuine murine model for RT, and specifically for AT/RT, is still lacking.

In this study, we have generated genetically modified mice that allow conditional inactivation of *Smarcb1*, by intercrossing Rosa26-Cre^ERT2^ (ref. 12) and *Smarcb1*^flox/flox^ mice^13^. Injection of tamoxifen at various time points allows a sequential inactivation of *Smarcb1* from early embryonic stages to adulthood. We show that the time for *Smarcb1* inactivation dramatically affects the phenotype. Moreover, we show that early embryonic inactivation of *Smarcb1* leads to a fully penetrant model of tumours, mainly intra-cranial, which faithfully resemble human RTs and recapitulate some of their diversity.

## Results

### *Smarcb1* inactivation in adult mice leads to lymphomas

**Figure 1:**
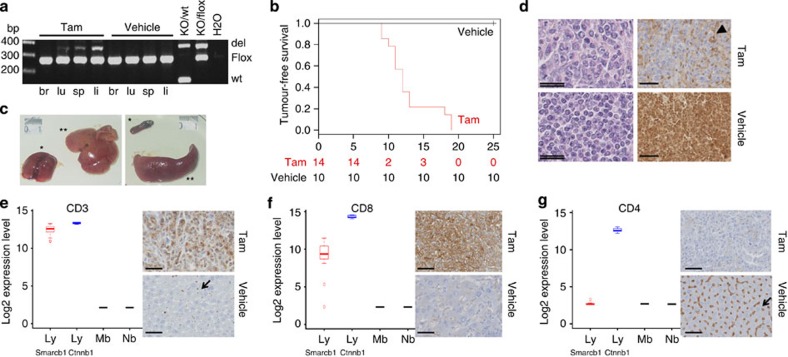
Mouse CD8(+) T-cell lymphomas. (**a**) PCR with primers specifically designed for the deleted (del), the undeleted (flox) and the wild-type (wt) alleles of *Smarcb1* in brain (br), lung (lu), spleen (sp) and liver (li); left panel: 5 days after injection of 2 mg per 20 g tamoxifen (Tam); right panel: mouse treated with vehicle. KO/wt: deleted and wild-type alleles from the *Smarcb1*^wt/del_ex2^ strain. KO/flox: deleted and flox alleles from *Smarcb1*^del_ex2/flox^ strain. (**b**) Tumour-free survival curve (Kaplan–Meier method) of *Smarcb1*^flox/flox^;Rosa26-Cre^ERT2^ when tamoxifen (tam) is injected at 8 weeks of age; time in weeks. (**c**) Macrospcopic aspect of liver (**, left panel) and spleen (**, right panel) in *Smarcb1*^flox/flox^;Rosa26-Cre^ERT2^ mouse when tamoxifen is injected at 8 weeks, as compared with liver and spleen from *Smarcb1*^flox/flox^;Rosa26-Cre^ERT2^ mouse treated with vehicle (*, left and right panels, respectively). (**d**) HES (left panels) and BAF47 immunostaining (right panels) on spleen from *Smarcb1*^flox/flox^;Rosa26-Cre^ERT2^ mouse treated with tamoxifen (upper panel) and vehicle (lower panel); tumour cells and loss of Baf47 expression are observed in tamoxifen treated organs only. The arrows show positive internal controls. Simple scale bars, 50 μm; double scare bars, 25 μm. (**e**–**g**) High expression of CD3 (**e**), high expression of CD8 (**f**) and low expression of CD4 (**g**) assessed by Affymetrix MOE430 2.0 arrays (left panels) and immunostaining (right panels). For histological sections, simple scale bars, 50 μm. CD3, CD8 and CD4 immunostainings are shown in livers from *Smarcb1*^flox/flox^;Rosa26-Cre^ERT2^ treated with tamoxifen (Tam, upper panels) or vehicle (lower panels). Arrows show normal lymphocytes (CD3 positive staining) and Kuppfer cells (CD4 positive staining) in control livers; the normal hepatic architecture is completely abolished in the CD8+/CD4− lymphomas. For Affymetrix data, expression in the *Smarcb1*-deficient tumours (Lymph_smarcb1_, *n*=7) are compared with medulloblastomas from the *Ptch1*^+/−^ model (Mb, *n*=4), neuroblastomas from the Th-Mycn model (Nb, *n*=3), and T-cell lymphomas from the *Ctnnb1*^del-ex3^ model (Lymph_ctnnb1_, *n*=7). In box plots, the central rectangle spans the first quartile to the third quartile (interquartile range or IQR); the horizontal line inside the rectangle shows the median; whiskers are taken to 1.5 × IQR from the quartile; circles show outliers.

Because the cell of origin of RT is unknown, we chose the ubiquitously active locus Rosa26 to drive Cre^ERT2^ expression and subsequent *Smarcb1* deletion. We first targeted *Smarcb1* by tamoxifen injection in adult mice (that is, 8 weeks). In agreement with Hameyer *et al*.^12^, we found an efficacious recombination in all tested organs except brains (Fig. 1a). As a result, we constantly obtained rapidly fatal tumours, massively infiltrating the liver and the spleen, in a median delay of 12 weeks (Fig. 1b,c); this phenotype was not observed in any mice treated with vehicle only (Fig. 1b–d). Consistently, Baf47 immunostaining was completely negative in the tumour cells, whereas its ubiquitous expression remains intact in normal contiguous cells (Fig. 1d) as in normal tissues of control mice (Fig. 1d). *Smarcb1* negative cell islets were also occasionally found in lungs and kidneys, but we did not observe any tumour in the CNS.

This phenotype, consisting of aggressive *Smarcb1*-deficient malignancy massively involving the spleen and the liver, was very much reminiscent of the mature CD8(+) T-cell lymphomas reported by Roberts *et al*. in *Smarcb1*^inv^;Mx1-Cre model^11^. To confirm this similarity, we compared the expression profiles of our tumours with a data set of non-*Smarcb1*-deficient tumours profiled on the same Affymetrix MOE430 arrays, consisting of Shh medulloblastomas (Mb, *Ptch1*^+/−^ model, provided by O.A.) and neuroblastomas (Nb, Th-Mycn model from our laboratory^14^); since lymphomas from Roberts' model were not analysed on the same array, we also aimed to compare our tumours with comparable, still different, lymphomas available from public data sets (CD8(+)CD4(+) lymphomas from ref. 15). Welch *t*-test-based comparisons followed by DAVID and Ingenuity Pathways Analyses highlighted the T-cell-like features of those tumours (Supplementary Fig. 1; Supplementary Data 1). More specifically, expression profiling and immunostaining confirmed that they strongly expressed CD3 (Fig. 1e) and CD8 (Fig. 1f), but did not express CD4 (Fig. 1g). Altogether, we concluded that targeting *Smarcb1* in adult mice leads to CD8(+)T-cell lymphomas, in full consistency with the CD8(+)T lymphomas previously described. However, as reported by Roberts *et al*., this model fails to give rise to any CNS tumour, which could at least be partly explained by a reduced recombination rate induced by Mx1-Cre (10% (ref. 16)) or Rosa26-Cre (Fig. 1a and ref. 12) in the adult brain.

### *Smarcb1* inactivation in neonates is lethal, without tumour

Given the early onset of RTs in humans and the failure to obtain RTs in adult mice, we hypothesized that targeting *Smarcb1* earlier in life may better mimic the actual clinical presentation of RTs. This first prompted us to inactivate *Smarcb1* in neonates. Semi-quantitative PCR in neonates treated with tamoxifen (Supplementary Fig. 2a) showed an actual recombination in all tested organs, including the brain and liver, yet with various efficacies.

**Figure 2:**
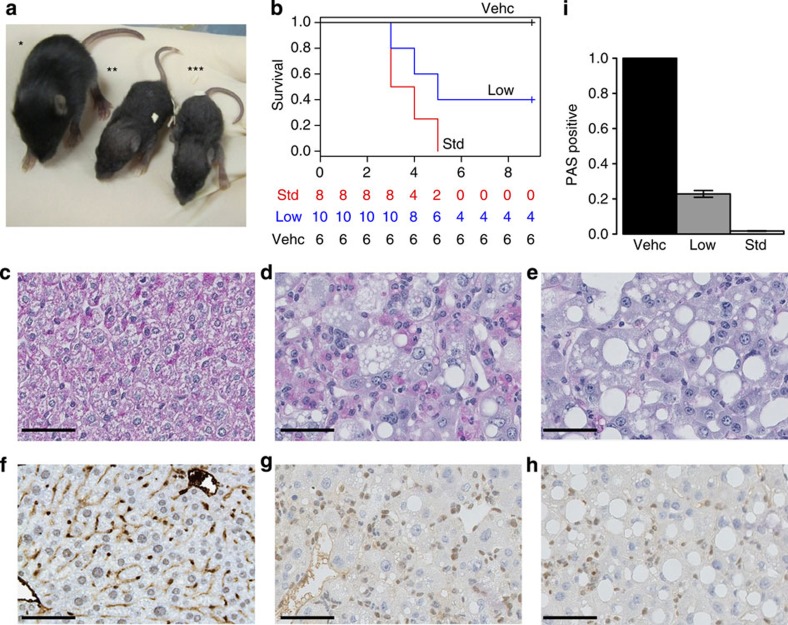
Phenotype of neonates upon *Smarcb1* inactivation. (**a**) Growth failure of mice treated with low doses (**) (1 mg per 20 g) and standard doses (***) (2 mg per 20 g) as compared with mice treated with vehicle (*) (**b**) Survival curve (Kaplan–Meier method) of neonates treated with vehicle (black line), low doses (blue line) and standard doses (red line) of tamoxifen. PAS (**c**–**e**) and Baf47 (**f**–**h**) staining on liver from mice treated with vehicle (**c**,**f**), low doses (**d**,**g**) and standard doses (**e**,**h**) of tamoxifen. (**i**) Quantification of the PAS staining in mouse treated with vehicle (vehc, *n*=6), low doses (low, *n*=6) and standard doses (std, *n*=6) of tamoxifen. The bars represent the s.e. Scale bars, 50 μm.

*Smarcb1* inactivation at P2-P3 led to severe growth failure (Fig. 2a) and constant rapid death, within the first month (Fig. 2b). However, extensive dissection of the mice did not show any tumour. In contrast, microscopic observation of liver sections showed massively vacuolized hepatocytes (Fig. 2c–h). In a context of specific hepatic *Smarcb1* deletion^13^, Gresh *et al*. previously reported a dramatically reduced glycogen storage in *Smarcb1*-deficient hepatocytes. Thus, we hypothesized that the hepatic phenotype we observed in our model could result from the same metabolic disturbances. Periodic Acid Schiff (PAS) staining confirmed that *Smarcb1*-deleted cells actually show reduced glycogen storage as compared with control mice (Fig. 2c–e,i). Other organs did not show obvious abnormality. Therefore, we assumed that a major failure in carbohydrate metabolism at least partly explained the death of those mice in short delays, which potentially precluded any tumour to develop. To circumvent this early lethality, we then used half doses of tamoxifen. Mice treated accordingly still showed growth failure and a high rate of death (60%) (Fig. 2b), at least partly related to the same, though mildly attenuated, hepatic phenotype (Fig. 2c–i). However, the 40% surviving mice did not develop any tumour (Fig. 2b).

### Reduced *Smarcb1* deletion prevents embryonic lethality

Based again on the early onset of RTs, with occasional antenatal and congenital presentations^17^, we finally postulated that inactivation of *Smarcb1* during the embryonic development may actually be relevant to induce RTs in mice. We therefore injected tamoxifen at standard doses (1.5–2 mg per 20 g) in pregnant mice from E1 to E18. In line with the early embryonic lethality of KO models previously reported^8^,^9^, no *Smarcb1* deleted pup was obtained when *Smarcb1* was inactivated between E1 and E5. When injected between E6 and E10, standard doses of tamoxifen constantly lead to early post-natal lethality of *Smarcb1*^flox/flox^;Rosa26-Cre^ERT2^ pups. We assumed that the lethality provoked by standard doses of tamoxifen during the earliest stages of embryonic development was due to an excessive deletion of *Smarcb1*, as reported previously^11^. However, we successfully circumvented the lethality provoked at the earliest stages by using lower doses of tamoxifen (Supplementary Fig. 3). As a result, our model allowed us to investigate the phenotypes associated with *Smarcb1* inactivation at various developmental stages, from E6 to E18.

### High penetrance and rapid onset of CNS tumours

**Figure 3:**
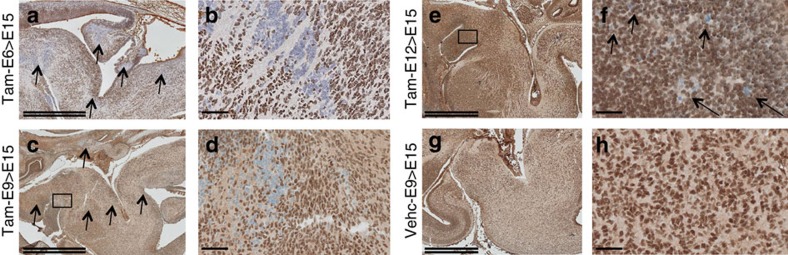
*Smarcb1*-deficient cells at E15 are scattered in the whole CNS. Baf47 immunostaining is performed at E15 on brains (centred on pons and posteriori fossa) from mice treated at (**a**,**b**) E6, (**c**,**d**) E9 and (**e**,**f**) E12 with tamoxifen (1 mg per 20 g); (**g**,**h**) control E15 brain after treatment with the vehicle at E9 (same results when the vehicle is injected at E6 and E12). Higher magnifications on the right panels focus on the *Smarcb1*-deficient cells; arrows depict *Smarcb1*-deficient areas or cells. Double scale bars, 1 mm; single scale bars, 50 μm.

We administered tamoxifen in pregnant mice from E6 to E18, using reduced doses of tamoxifen from E6 to E12 and normal doses from E12 to E18 (Supplementary Fig. 3). As a result, the fraction of living pups was normal at all stages. By semi-quantitative PCR and using Rosa26-LacZ reporter strain, we first demonstrated that the recombination was ubiquitous at all stages, though in variable degrees (Supplementary Fig. 2). In particular, *Smarcb1* locus was recombined in the brain at all stages, but with a higher efficacy before E12 (Supplementary Fig. 2a,b). Baf47 immunostaining nevertheless clearly showed *Smarcb1*-deficient cells scattered in the CNS without specific anatomical pattern, when tamoxifen was injected at E6, E9 or E12 (Fig. 3).

**Figure 4:**
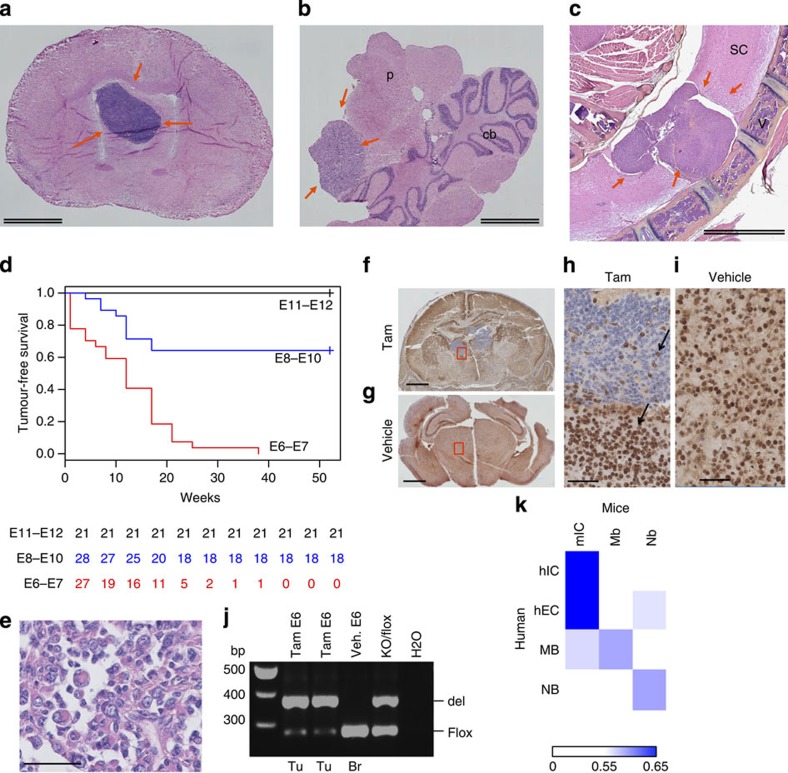
Characteristics of mouse *Smarcb1-*deficient tumours. Macroscopic dissection of the CNS in *Smarcb1*^flox/flox^;Rosa26-Cre^ERT2^ showing tumours. (**a**) In the sub-ependymal region, (**b**) in the ponto-cerebellum angle, (cb, cerebellum; p, pons) and (**c**) in the spinal cord (v, vertebra; sc, spinal cord). Double scale bars, 2 mm. (**d**) Tumour-free survival after tamoxifen injection (0.5–1 mg per 20 g) at E6–E7, E8–E10 and E11–E12; time in weeks. Numbers are reported under the curve for each category. (**e**) Rhabdoid phenotype of tumour cells (hematoxylin eosin safran staining): prominent nucleoli, uncondensed nuclei, eosinophilic cytoplasmic inclusions. (**f**–**i**) Baf47 immunostaining; low magnification of a coronal section at P2 of brains in *Smarcb1*^flox/flox^;Rosa26-Cre^ERT2^ mice treated at E6 with tamoxifen (Tam) (**f**) and vehicle (**g**); higher magnification of the same coronal sections (**h**,**i**). Areas with negative staining are seen in mice treated with tamoxifen only; normal internal controls stain positively (arrows). Single scale bars, 50 μm. (**j**) PCR with primers specifically designed for the deleted (del) and undeleted (flox) alleles of *Smarcb1;* Tu, 2 different brain tumours veh, vehicle; KO/flox, control DNA from of a *Smarcb1*^del_ex2/flox^ mouse; (**k**) AGDEX correlation score performed on all ortholog genes comparing a set of mouse and human tumours. The reference tissues used for the comparisons were normal cerebral cortex in both species. Matched control tumours: human *SHH* medulloblastomas (MB) and mouse medulloblastomas (Mb, *Ptch1*^+/−^ model); human *MYCN*-amplified neuroblastomas (NB) and mouse neuroblastomas (Nb, Th-Mycn model).

Strikingly, inactivation of *Smarcb1* at E6-E7 almost constantly resulted in intra-cranial (hippocampus region, cerebellum and cerebral cortex) (Fig. 4a,b) or spinal tumours (Fig. 4c), revealed by neurological defects (gait disturbance or paraplegia), in a median delay of 90 days (Fig. 4d). One tumour only arose from a paw, in the soft parts. The tumour penetrance decreased when tamoxifen was administered from E8 to E10 (Fig. 4d); it became null from E11 to E18 (Fig. 4d).

### *Smarcb1*-deficient mouse tumours resemble human RTs

The histological analysis of those tumours showed small round undifferentiated features, resembling human primitive neuro-ectodermal tumour, or cytoplasmic eosinophilic inclusions, uncondensed chromatin and prominent nucleoli, highly suggestive of rhabdoid features (Fig. 4e). As expected, all tumours showed a complete loss of *Smarcb1* expression, while vehicle-treated brains showed a homogenously positive staining (Fig. 4f–i). We confirmed that the loss of Baf47 staining was actually due to a deletion of the floxed allele in all tumours tested (Fig. 4j). Hence, histological and genetic patterns were very similar to human RTs.

To address the relevance of these mouse tumours for human diseases, we profiled a set of 50 human RTs including 30 intra-cranial (hIC) and 20 extra-cranial (hEC) tumours from various locations (clinical annotation summarized in Supplementary Data 1) and compared these expression profiles with those of mouse tumours using AGDEX algorithm designed for cross-species comparison^18^. We first calculated AGDEX scores of human SHH medulloblastomas and *MYCN*-amplified neuroblastomas with mouse medulloblastomas (*Ptch1*^+/−^ model) and mouse neuroblastomas (Th-Mycn model), respectively. These are two well-established models known to recapitulate their human counterparts: AGDEX scores were 0.58 and 0.59, respectively (Fig. 4k). Interestingly, *Smarcb1*-deficient intracranial tumours from our model showed even higher AGDEX correlation scores when compared with hIC and hEC (0.67 and 0.68, respectively), confirming their relevance to human RTs (Fig. 4k).

### Diversity within mouse *Smarcb1*-deficient tumours

**Figure 5:**
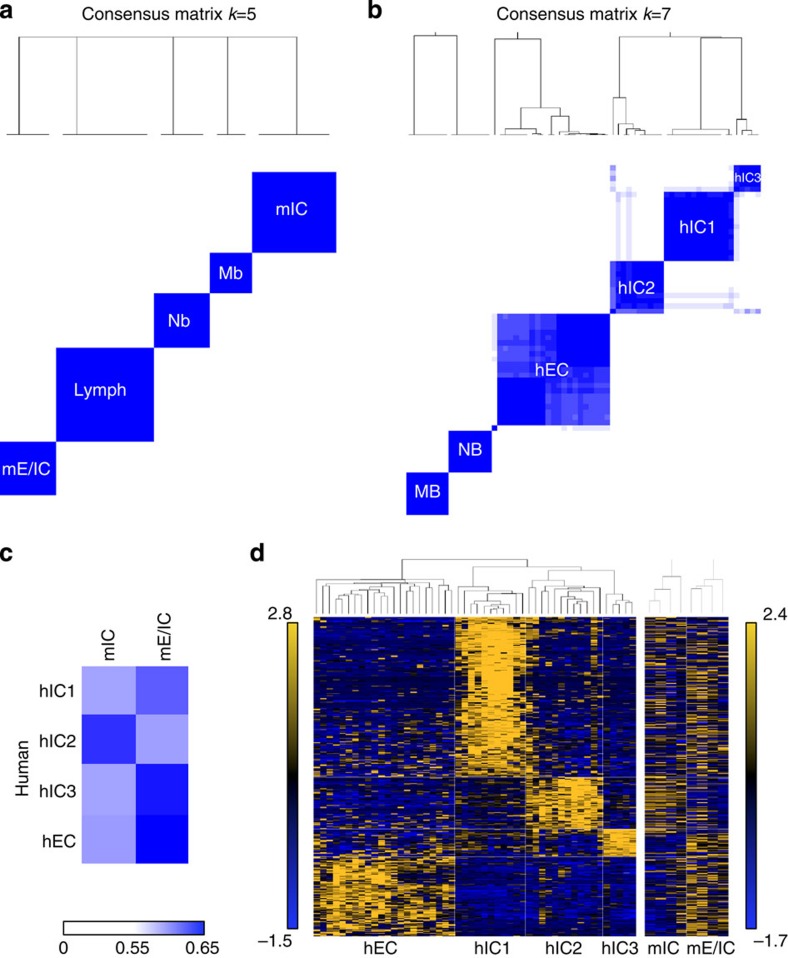
Mouse CNS tumours reflect the diversity of human RTs. (**a**) Differential subgroups were identified using ConsensusClusterPlus R package v1.18.0 (with 1,000 resamplings, average linkage hierarchical clustering algorithm and Pearson correlation distance), based on the expressed and variable genes obtained after elimination of background (threshold: 1.2) and invariant genes using RIQR (threshold: 0.75; max(Q3−Q2,Q1−Q_2_)/Q_2_). Mb, mice medulloblastomas (*Ptch1*^+/−^ model, *n*=3); Nb, mice neuroblastomas (Th-Mycn model, *n*=4); lymph, CD8(+) T-cell lymphomas (*n*=7); mIC, murine intra-cranial tumours (*n*=5); mE/IC (*n*=4). (**b**) Differential subgroups among human tumours were identified by ConsensusClusterPlus R package, as aforementioned. hIC group (human intracranial) consists of 28/28 intracranial tumours and is dissected in three subgroups (hIC1, hIC2 and hIC3). hEC group (human extracranial tumours) consists of 20/22 extracranial tumours and 2/22 intracranial tumours. MB: medulloblastomas, SHH subtype; NB: *MYCN*-amplified neuroblastomas. (**c**) AGDEX score performed on each mouse and human subgroups. (**d**) Left panel: clustering on a set of 540 genes (268+98+42+132) whose higher expression levels specifically define hEC, hIC1, hIC2 and hIC3, respectively; the list is obtained by pair-wise Welch *t*-test (*P*≤0.05) analyses, and limited to genes with a fold change |FC|≥1.2 (log2 expression value). Right panel: clustering on the two mouse subgroups using the ortholog genes.

To further characterize those mouse tumours, we applied three orthogonal unsupervised clustering methods: unsupervised hierarchical clustering, consensus clustering and non-negative matrix factorization (NMF). We thus compared all *Smarcb1-*deficient (from *Smarcb1*^wt/del_ex1-2^ and *Smarcb1*^flox/flox^;Rosa26-Cre^ERT2^ models, that is, lymphomas, intracranial tumours and extracranial tumours) and non *Smarcb1*-deficient mouse tumours aforementioned (medulloblastomas and neuroblastomas). The hierarchical unsupervised clustering (Supplementary Fig. 4a) distinguished two main branches (i) the first one consisting of three subgroups made of medulloblastomas, neuroblastomas and a subset of intracranial *Smarcb1*-deficient tumours (a group hereafter referred to as mIC), and (ii) the second one consisting of two subgroups, one made of *Smarcb1*-deficient lymphomas only, and the last one made of the two extracranial tumours and the remaining intracranial tumours (a group hereafter referred to as mE/IC). Consistently, a Pearson correlation matrix demonstrated that half of the intracranial tumours correlated with extracranial tumours but did not correlate at all with the other half of intracranial tumours (Supplementary Fig. 4b). To confirm those results, we next performed NMF (Supplementary Fig. 4c) and consensus clustering (Fig. 5a; Supplementary Fig. 4d) on the same data set, Mb and Nb serving as controls for truly existing groups. Thereby, the optimal *k* value that allows splitting Mb and Nb also defined three tumour groups within *Smarcb1*-deficient tumours: CD8(+)T-cell lymphomas and two other subgroups (Fig. 5a). One consisted exclusively in intracranial tumours (corresponding to mIC), and the other consisted in the two extracranial tumours and the remaining intracranial tumours (corresponding to mE/IC). Altogether, these results converge to split our intracranial tumours in two distinct entities, one clustering with extracranial *Smarcb1*-deficient tumours, and the other clustering close to tumours of neuronal origin, that is, medulloblastomas and neuroblastomas.

Comparisons of mIC and mE/IC, followed by DAVID analyses, revealed that mIC was characterized by a significant over-representation of neuronal development pathways (Supplementary Data 2). In contrast, mE/IC were characterized by few referenced pathways or gene networks. None referred to brain development or neuron differentiation.

### Comparable diversity in mouse and human tumours

The diversity observed within mouse tumours prompted us to investigate whether human RTs also show similar heterogeneity. With this aim, we performed the same three parallel clustering methods on the 50 aforementioned human RTs. In line with the subgroups observed in mouse, unsupervised hierarchical clustering of transcriptome profiles in humans also delineated three subgroups within hIC (hIC1, hIC2 and hIC3, Supplementary Fig. 5a). NMF (Supplementary Fig. 5b) and consensus clustering (Fig. 5b; Supplementary Fig. 5c) both determined that the optimal *k* value was *k*=6, also dissecting hIC in the three subgroups identified by hierarchical clustering. Pair-wise differential analyses defined a specific genes signature for each group (Supplementary Data 3). DAVID analysis on these sets of genes confirmed the neuronal pattern for all hIC sub-groups when compared with hEC (Supplementary Data 3); conversely, this latter group was characterized by markers of embryonic morphogenesis and skeletal development.

We then further investigated whether mouse tumour subgroups preferentially recapitulate one human group or another, using AGDEX scores. High scores were observed between mIC and hIC2, and mE/IC and hEC (Fig. 5c). We then identified lists of genes specifically overexpressed in each human group by pair-wise Welch *t*-test; clustering mouse tumours on this set of genes also clearly delineated mIC from mE/IC and showed similarities between mIC and hIC2 patterns (Fig. 5d).

### Expression profiles suggest distinct cells of origin

**Figure 6:**
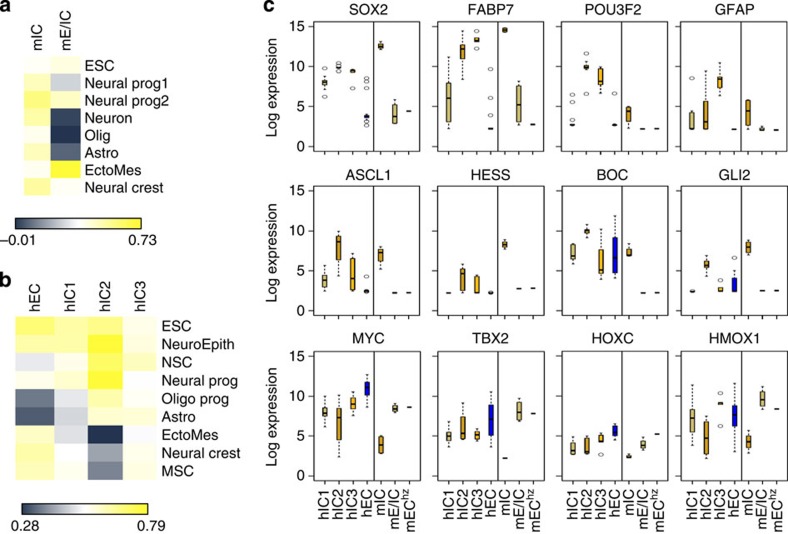
Transcriptome profiles suggest various cells of origin for human and mice *SMARCB1*-deficient tumours. (**a**) Pearson correlation between transcriptome profiles of mIC and mE/IC from one part, and various mouse embryonal tissues from the other part. ESC: embryonic stem cell (GSE44175). Neural Prog 1, neural progenitor (GSE44175); neural Prog 2, neural progenitor (GSE44175); neuron (GSE46150); olig, oligodendrocytes derived from neural stem cells (GSE9566); astro, astrocytes derived from neural stem cells (GSE9566); EctoMes, ectomesodermal tissue from the palate (GSE9566); neural crest (GSE11149). (**b**) Pearson correlation between trancriptome profiling of hICs and hEC from one part, and various embryonal tissues from the other part. ESC (GSE55679); NeuroEpith, NeuroEpithelium (GSE55679); NSC, neural stem cell obtained from foetal cortex (GSE15209); neural Prog (GSE56906); glial Prog, glial progenitors (GSE36634); oligo Prog, adult oligodendrocytes progenitors (GSE29368); astro, astrocytes (GSE18959); MSC, mesenchymal stem cells (GSE37603); neural crest (GSE14340); EctoMes (GSE24598). (**c**) Log2 expression of genes obtained on Affymetrix U133Plus2.0 and MOE430 arrays. Human genes are plotted on the left panels, mouse orthologs on the right panels. hIC1, *n*=11; hIC2, *n*=;12 hIC3, *n*=5; hEC, *n*=20; mIC, *n*=5; mE/IC, *n*=4; mEC^Hz^ refers to the face tumour obtained from the heterozygous *Smarcb1*^wt/del_ex1-2^ model, as a ‘standard' mouse rhabdoid tumour (*n*=1). In box plots, the central rectangle spans the first quartile to the third quartile (interquantile range or IQR); the horizontal line inside the rectangle shows the median; whiskers are taken to 1.5 × IQR from the quartile; circles show outliers. For HOXC cluster, the mean expression of all genes in the cluster is plotted as one single value. *SOX2*, *FABP7* and *POU3F2* are co-expressed in neural stem cells; *ASCL1* and *HES5* exert antagonistic effects in neural stem cells to influence the lineage commitment; *BOC* and *GLI2* signals the SHH pathway in neural stem cells; *TBX2* and *HMOX1* are homeobox genes expressed by mesenchymal cells.

The developmental window comprised between E6 and E10 is characterized by the commitment of stem cells to specified progenitors. We therefore postulated that *Smarcb1* inactivation at E6-E10 may target such stem or progenitor cells, as cells of origin of the tumours. To get insights in which cells gave rise to which mouse tumours, we correlated transcriptome profiles from our study with transcriptome profiles from stem or progenitor cells from the cephalic region at E6 to E10 in publicly available databases (embryonic Stem Cell (GSE44175), neural progenitor (GSE44175 and GSE44175), neuron (GSE46150), oligodendrocytes derived from neural stem cells (GSE9566), astrocytes derived from neural stem cells (GSE9566), ectomesodermal tissue from the palate (GSE9566) and neural crest (GSE11149)). As shown in Fig. 6a, the highest correlation for mIC was obtained with neuron progenitors, while mE/IC best correlated with ectomesenchyme, a cephalic neural crest cell-derived mesoderm.

We then similarly compared human tumours with human stem or progenitor cell profiles available in public databases (Fig. 6b). The most striking correlation was found between hIC2 and neuro-epithelium, neural stem cells and neuron progenitors; hIC1 and hIC3 showed weaker correlations with these early neuronal lineages. Conversely, high correlation was observed between hEC and embryonic stem cells as well as neural crest-derived cells.

To corroborate these correlations based on genome-wide expression, we looked in more detail at the expression pattern of stem or progenitor cell-specific markers, both in mouse and human tumours. hIC2 and mIC were both characterized by the overexpression of *SOX2*, *POU3F1/2* and *ASCL1*, a combination of genes expressed in neural stem cells and capable of reprograming fibroblasts into neurons^19^,^20^ (Fig. 6c, Supplementary Data 2 and 3). They also overexpress *HES5*, a Notch effector antagonizing the pro-differentiating effects of *ASCL1* in neural stem cells at E8-E8.5 (refs 21, 22, 23), *FABP7*, an intracytoplasmic marker of neural stem cells^24^, and components of the SHH pathway (*BOC*, *GLI2*) (Fig. 6c and Supplementary Fig. 6). mIC was also characterized by a high expression of *Mycn*, which was completely absent from mE/IC tumours (Supplementary Data 2).

In contrast, hIC1 were characterized by a lower expression of *SOX2* and *FABP7*, and absence of expression of *POU3F2*, *ASCL1*, *HES5* (Fig. 6c; Supplementary Fig. 6; Supplementary Data 3). However, they strongly expressed *OTX2* and *ODZ2*, two markers of neural progenitors^25^,^26^ (Supplementary Data 3). They also showed higher expression of BMP pathway genes, such as BMP4, BAMBI and SMAD7 (Supplementary Data 3). hIC3 expressed a combination of genes suggesting neural stem cells (*POU3F2*, *SOX2*, *FABP7*), but also radial glial (*GFAP*)^27^ commitment (Fig. 6c; Supplementary Fig. 6; Supplementary Data 3).

All hIC groups showed a high expression of *ACTL6A* and no expression of *ACTL6B*, suggesting that these tumours did not yet commit the switch from the ‘neural progenitor' to the ‘neural' BAF complex^28^. The same pattern was found in mIC (Supplementary Fig. 7).

Finally, hEC showed no expression of these neural genes but overexpressed homeobox genes critically involved in early development of mesodermal-derived structures; among those homeobox genes, *TBX2* (ref. 29) and *HOXA/C* genes^30^,^31^ (Fig. 6c; Supplementary Fig. 6) were also found overexpressed in mE/IC. hEC and mE/IC both showed a distinctive expression of TGFB pathway (*TGFBR2*, *TGFBR3*, *SMAD7*). Finally, hEC and mE/IC were also characterized by an overexpression of *MYC* oncogene, which was virtually absent from mIC (Fig. 6c).

## Discussion

Our data first show that the developmental stage targeted by *Smarcb1* inactivation dramatically impacts the type of tumours obtained. Adult mouse tumours in our study are very similar to those published by Roberts *et al*.^11^. These authors used the Mx-Cre system to drive an inducible inversion of *Smarcb1* on interferon or polyI/polyC administration. Our model emphasizes that the occurrence of lymphomas is not due to the Interferon/Mx-Cre system, which might preferentially target liver, spleen or hematopoietic cells^16^. In contrast, the convergence of these two models reveals that T cells are exquisitely sensitive to *Smarcb1* deletion in adult mice. Wang *et al*.^32^ showed that a highly specific population of T cells, for example, mature memory CD8(+)CD44^high^CD122^low^ cells, was able to undergo a malignant transformation on *Smarcb1-*deletion. The absence of these cells in neonates and their expansion throughout life and maturation of the immune system^33^ fit with a temporally restricted proneness to developing this type of lymphomas during post-natal life.

This lymphoma phenotype is, however, fundamentally different from any *SMARCB1*-deficient tumours observed in Humans. Face and neck tumours obtained from heterozygous knockout of *Smarcb1* have been suggested to constitute a good mouse model for human extra cerebral RTs. Unfortunately, due to the low penetrance of this model (1/10), we could only include one such tumour in our comparisons of expression profilings. Nevertheless, the co-clustering in a single mE/IC group of the face tumour from the heterozygous model and of intra- and extracranial tumours from our inducible model suggests that the two models lead to similar types of tumours. The assumption that these models recapitulate human extracranial RTs is further corroborated by the high AGDEX correlation obtained between mE/IC and hEC groups.

Our highly penetrant, AT/RT-like, mIC group brings major novelties among previously described *Smarcb1*-deficient mouse tumours (summarized in Supplementary Table 1). Dependent on *Smarcb1* inactivation only, they truly mimic human RTs' oncogenesis. Furthermore, they present strong correlation with hIC2; as such, they now offer a faithful model for at least a subset of AT/RTs.

Inactivating *Smarcb1* between E6 and E10 provokes a *Smarcb1* loss necessarily in embryonic stem cells or progenitors, which are predominant at that time. In the cephalic region at these early developmental stages, the neuroepithelium of the closing neural tube is giving birth to neural stem cells and cephalic neural crest cells. The high correlation between mE/IC and ectomesodermal tissue may suggest cephalic neural crest-derived cells as cells of origin for this group, in consistency with their AGDEX homology with the hEC group, the expression profile of which also correlates with this neural crest cells or MSCs. In contrast, mIC show higher correlation with neural progenitors, which may thus be their cell of origin; this is fully consistent with hIC2 also showing strong correlations with neural stem cells and neural progenitors. Both mIC and hIC2 harbour high levels of *SOX2*, *ASCL1*, *POU3F1/2* and *HES5*, genes involved in the fine-tune regulation of neural stem cells commitment in neuronal progenitors. Since the integrity of the SWI/SNF complex is required for the commitment of neuron stem cells into neuronal progenitors^34^,^35^, it may be speculated that targeting *Smarcb1* from E6 to E10 in the CNS impairs the progression of neural stem cells through neural differentiation, which may constitute a critical step in oncogenesis.

The unexpected heterogeneity within hIC tumours may then reflect that various stem or progenitor cells are able to survive to *Smarcb1* inactivation, and subsequently give rise to significantly different tumours. Torchia *et al*.^36^ recently published expression profiling on a series of 51 AT/RT that corroborate our results. They indeed identified a first group of AT/RT that was characterized by higher expressions of *ASCL1* and *HES5* (NOTCH pathway), *FABP7*, *POU3F2*, *SOX2* and *MYCN* genes; all those features are reminiscent of our mIC and hIC2 groups and accordingly strongly suggesting a neural origin. In contrast, the second group identified by Torchia *et al*. was characterized by a higher expression of genes involved in the BMP pathway (*BM4*, *BAMBI*, *SMAD6*), *CLIC6*, *OTX2* and *MSX1*, all features they describe as ‘mesenchymal' and which are also characteristic of our hIC1 group. Interestingly, they propose two kinds of cells of origin for AT/RT, one deriving from neural progenitors, and the other having more mesenchymal lineage markers, a conclusion fully consistent with the hypotheses coming from our mouse model.

Of note, our study and Torchia's series both focus on paediatric AT/RT, while a few AT/RT adult cases are reported^37^, challenging the proposed hypotheses of early embryonic progenitors as exclusive cells of origin. One explanation could be that a pool of embryonic neuron stem cells remains until adulthood. Alternatively, one could also hypothesize that adult AT/RT are truly different from their paediatric counterparts, as it was previously published for adult and paediatric medulloblastomas^38^. The observation of neuroglial tumours with *BRAF* V600E mutation acquiring a AT/RT-like phenotype on *SMARCB1* loss^39^ illustrates that some cells with abnormal genetic background may tolerate *SMARCB1* loss and undergo malignant transformation with a rhabdoid phenotype, without being actual AT/RT, restrictedly defined as an infant disease characterized by a remarkably simple genome.

Our results suggest that a restricted early developmental window is required to most efficaciously target the putative pool of cells of origin for AT/RT. Although the lower recombination rate in brain after E10 may influence penetrance, *Smarcb1*-deficient cells can be observed at E12 but cannot give rise to tumours. Such an observation is consistent with the lack of tumour phenotype in *Smarcb1*^flox/flox^;Atoh1-Cre model^40^; this model demonstrates that *Smarcb1*-deficient granule cell precursors fail to develop and lead to severe cerebellum hypoplasia, indicating that AT/RT do not arise from granule cell precursors but from a distinct, developmentally restricted cell population. These important results encourage developing now new conditional *Smarcb1*^flox/flox^ mouse models, using Cre^ERT2^ under the control of promoters specific for each cell type or developmental stage, such as *SOX2* (stemness features), *ASCL1* (progenitors at the lateral inhibition step), *OTX2* (neuronal progenitors) or *MSX1* (ectodermal progenitors) to validate our hypothesis. We may then be able to more accurately determine which kind of progenitor can actually tolerate *Smarcb1* depletion and give rise to the various AT/RT subgroups.

The metabolic failure observed in neonates also exemplifies the time-dependent phenotypes obtained on *Smarcb1* loss. The increasing recombination rates during the liver development (Supplementary Fig. 2a) as well as the survival of 4/10 neonates when using half doses of tamoxifen (Fig. 2b) demonstrated that the variation of phenotypes from E12 to E18 (no death) and after birth (high mortality) might tightly depend on the percentage of remaining *Smarcb1*-proficient hepatocytes; presumably also, the maternal liver may supplement a transient foetal liver deficiency until birth. However, the total absence of tumours within the few surviving neonates, neither in the brain nor elsewhere in the body, again argues in favour of a time-restricted phenotype. Neonates may lack both the sufficient pool of neural stem cells to develop AT/RT-like tumours, and the mature memory lymphocytes to develop CD8+ lymphomas.

Finally, our model recapitulates the diversity of human RTs in a much broader way than previously published models. In particular, this model offers the first highly penetrant model for intracranial tumours. Interestingly, mIC and hIC2 are both characterized by genes involved in SHH signalling, which is consistent with previous reports linking *SMARCB1* loss to *GLI1* overexpression^41^. This observation might be of clinical relevance, given the potential sensitivity of some AT/RT cell lines to SHH inhibitors^42^. In that perspective, our model not only brings new insights on the developmental biology underlying RTs oncogenesis; it also offers encouraging conditions for preclinical treatment of this harmful disease.

## Methods

### Animals

For *Smarcb1*^flox/flox^ mouse strain, exons 1 and 2 of *Smarcb1* gene were flanked by two loxP sites^13^; this strain was crossed with a knock-in mouse line harbouring the Cre^ERT2^ coding region under the control of the Rosa26 locus (Rosa26-Cre^ERT2^)^12^. Mice were genotyped according to these references for *Smarcb1* del/flox/wt PCR: forward1: 5′-CTTGCCAGGTGAGCAGTCTG-3′, forward2: 5′-GTTGTTAGTCCCTTTGCTCC-3′, reverse: 5′-GCCACCAGCCAGATGTCATAC-3′. PCR product sizes were: wild-type allele: 150 bp; floxed allele: 250 bp; deleted allele: 400 bp (Supplementary Fig. 8). All experiments were performed on mixed background (129/SV × C57BL/6). The sex ratio within groups was in equilibrium. Protocol and animal housing were in accordance with national regulation and international guidelines^43^. Approval for this study was received from the Institutional CEST review board (Comite d'Evaluation et de Suivi de Recherche Translationnelle) from Curie Institute, and from the Direction Generale de la Recherche et de l'Innovation, Ministere de l'Enseignement Superieur et de la Recherche (authorization number 6,150).

We use PGK-Cre mice (from Curie animal facility) to get *Smarcb1*^wt/del_ex1-2^: Cre is driven by early acting PGK-1 promoter, the recombinase is under dominant maternal control^44^.

*Smarcb1*^wt/del_ex1-2^ mice were generated by crossing PGK-Cre female with *Smarcb1*^flox/wt^ male. Ten *Smarcb1*^wt/del_ex1-2^ mice (5 males and 5 females, adults) were followed. *Smarcb1*^del_ex1-2/flox^ mice were generated by crossing *Smarcb1*^flox/flox^ with Smarcb1^wt/del_ex1-2^.

The Rosa26-LacZ reporter strain was previously published in Sariono *et al*.^45^.

### Tamoxifen administration

A single dose of tamoxifen (Sigma, T5648, dissolved at 10 mg ml^−1^ in sterile ethanol:sun flower oil 1:10) was administrated *per os* to pregnant *Smarcb1*^flox/flox^; Rosa26-Cre^ERT2^ mice at 6, 7, 8, 9, 10, 11, 12, 15 and 18 days post coitum (post-coital plug observation defined day 0) as shown in Supplementary Fig. 3. All foetuses were delivered by caesarean section (E19) and raised by foster mothers. Neonate (P2 or P3) and adult *Smarcb1*^flox/flox^;Rosa26-Cre^ERT2^ mice were injected intraperitoneally with a single conventional dose of tamoxifen (2 mg per 20 g mice). A second cohort of neonates was treated with a single reduced dose of tamoxifen (1 mg per 20 g mice) at P2 (Supplementary Fig. 3).

Five to ten *Smarcb1*^flox/flox^;Rosa26-Cre^ERT2^ mice were treated with the vehicle (sunflower oil) at all stages of development, in embryos (E6, E9, E12- not later on, since no phenotype was observed in embryos treated with tamoxifen after E12), neonates and adults. R26-Cre^ERT2^; Rosa26-LacZ gestant females were treated with tamoxifen (1 mg per 20 mg) at E6 and E9, LacZ staining was analysed at E15 (*n*≥5 embryos for each point).

### Histological examination

Organs were collected, frozen on dry ice and processed for cryosectionning and macro-dissection) or fixed in AFA (Carlo Erba, ref: 526263001) for histological examination. BAF47 immunohistochemistry was performed on fixed paraffin-embedded tissue using BD, code 612111, clone 25/BAF47, dilution 1/50 (ref. 46). CD3 (Dako, code IS503, dilution 1/200), CD4 (Dako, code IS649, clone 4B12, dilution 1/200) and CD8 (Dako, code IS623, clone C8/144B, dilution 1/200) were carried out utilizing commercially staining kit (Dako Envision Kit, K4009 for CD3 and Vector laboratories mouse on mouse basic kit for CD4 and CD8).

PAS staining was performed on fixed paraffin-embedded neonate liver as described in ref. 13. Quantifications were done for PAS by counting the number of positive areas at magnification × 10, in eight livers for 2 mg per 20 g group and six livers for 1 mg per 20 g group.

Frozen sagittal section β-galactosidase stainings (Supplementary Fig. 2) were performed as described in Echelard *et al*.^47^, on embryos freshly taken out (E6, E9, E12) at E15 and on neonates.

### Mouse tumours macrodissection and RNA extraction

Frozen brains were serially sectioned using a cryostat at 4 μm; quick Hematoxilin stainings were performed on each section until a tumour could be identified; macrodissection was then performed with a sterile scalpel. Small pieces of tissue containing the tumour cells were frozen at −80 °C until RNA preparation. The tumour RNAs were extracted using a miRNeasy mini kit (Qiagen ref: 217004).

### Human samples selection and RNA extraction

Human RTs were referred to our Institute for assessment of *SMARCB1* gene status. We selected tumours only when the diagnosis was made before 5 years of age. BAF47 immunohistochemistry was assessed as described in ref. 46 and *SMARCB1* molecular analysis was performed as described in ref. 48. Samples were used for research purposes according to French Huriet Law regarding research on human tissues. Total RNAs were obtained from frozen samples using Qiagen QIAamp RNAeasy kit, according to the manufacturer's procedures. The tumour cell content was visually estimated before RNA extractions; tumour cell content lower than 60% was an exclusion criteria. Samples were used according to the French Hurriet law (88–1,138) regarding research on human tissues. Frozen samples were provided by French tumour banks from consenting patients. Approval for this study was received from the Institutional CEST review board (Comite d'Evaluation et de Suivi de recherche Translationnelle) form Curie Institute.

### Quantitative RT-PCR

Reverse transcription was carried out on 0.5 μg total RNA from human and mouse tumours with oligo(dT) primers following the manufacturer's instructions (High capacity cDNA Reverse Transcription Kit, Applied Biosystems). Expression levels of genes were analysed using real-time, quantitative PCR. All amplifications were done using the SYBR Green PCR Master Mix (Applied Biosystems) and the CFX384 machine (Bio-Rad). The relative quantification for gene expression was determined using the 2^−ΔΔCt^ method. The TBP gene was used as internal control for data normalization. Primers used in this publication are available in Supplementary Table 2.

### Microarray analysis

Micro-array data have been deposited in the GEO database under accession code GSE64019.

In *Smarcb1*^flox/flox^;Rosa26-Cre^ERT2^ mice, transcriptomes of seven *Smarcb1*-deficient lymphomas, nine *Smarcb*1-deficient intracranial tumours and one soft-tissue tumour were analysed. From the *Smarcb1*^delx_ex2/wt^ mouse model, only one face tumour was analysed. The transcriptomes of seven *Ctnnb*^del_ex3^ lymphomas (GSE7050), three neuroblastomas (previously published, GSE46583)^14^ and four medulloblastomas were analysed. In humans, 20 extra-CNS RTs, 30 intra-CNS RTs, 8 SHH medulloblastomas and 8 *MYCN* amplified neuroblastomas were profiled. cDNA from murine tumours were hybridized to Affymetrix Murine MOE430 2.0 arrays, while human samples were hybridized to Affymetrix U133Plus2.0 arrays. Gene expression data was normalized using gcRMA algorithm on custom Brainarray CDF^49^.

Differential subgroups were defined using different methods: unsupervised hierarchical clustering (average linkage and Pearson correlation distance), consensus clustering (using ConsensusClusterPlus R package v1.18.0, with 1,000 resamplings, average linkage hierarchical clustering and Pearson correlation) and NMF (using NMF R package, with standard NMF algorithm method performed on 30 runs). The optimal number of subgroups have been defined on the lower proportion of ambiguous clustering value (PAC*k*= CDF*k*(valueindex(max))−CDF*k*(valueindex(min))) and biological knowledge for consensus clustering method; and on the better cophenetic correlation score and biological knowledge for NMF method. All these methods are based on the expressed and variable genes obtained after elimination of background (threshold: 1.2) and invariant genes using RIQR (threshold: 0.75; max(Q3−Q2,Q1−Q3)/Q2).

Differentially expressed genes between two groups were defined using Welch *t*-test (*P*≤0.05 adjusted with Benjamini and Hochberg) and fold change (|FC|≥1.2).

Concordance of orthologous genes between human and mouse was made with HomoloGene.

For intraspecies comparisons, Pearson correlation score on transcriptome profilings were calculated by correlating the means of each gene expression value, after filtering those below the background and unvariant genes.

The agreement between human and mouse gene expression tissues was evaluated using AGDEX (Agreement of differential expression) R package v.1.6.0, based on a statistical procedure described in Johnson *et al*.^18^. The reference tissues were prefrontal cortex—downloaded from the GEO database (GSE11512 for human, GSE16660 for mice). Comparisons between human *MYCN*-amplified neuroblastomas (previously published, GSE12460) and murine neuroblastomas from the Th-Mycn model (previously published, GSE46583) on one hand, and SHH medulloblastomas in humans^50^ (previously published, GSE12992) and shh medulloblastomas from *Ptch1*^+/−^ models, on the other hand, served as controls. All bioinformatic analyses were performed with R software environment.

## Additional information

**Accession codes:** The micro-array data have been deposited in the GEO database under accession code GSE64019.

**How to cite this article:** Han, Z.-Y. *et al*. The occurrence of intracranial rhabdoid tumours in mice depends on temporal control of *Smarcb1* inactivation. *Nat. Commun.* 7:10421 doi: 10.1038/ncomms10421 (2016).

## Supplementary Material

Supplementary Information Supplementary Figures 1-8 and Supplementary Tables 1-2.

Supplementary Data 1 Main annotations of human tumours.

Supplementary Data 2 Comparison between mIC and mE/IC (mIC vs mEIC)

Supplementary Data 3 Comparison between mIC and Mb (mIC vs Mb)
