# Supplementary material for: The occurrence of intracranial rhabdoid tumours in mice depends on temporal control of Smarcb1 inactivation
Source: Nat Commun. 2016 Jan 28;7:10421. doi: 10.1038/ncomms10421 (PMC4738337; doi:10.1038/ncomms10421)
Supplement: Supplementary Information — Supplementary Figures 1-8 and Supplementary Tables 1-2. [file ncomms10421-s1.pdf]

Supplementary Figure 1. Biological characteristics of *Smarcb1*$^{flox/flox}$; Rosa26-Cre^{ERT2}; lymphomas

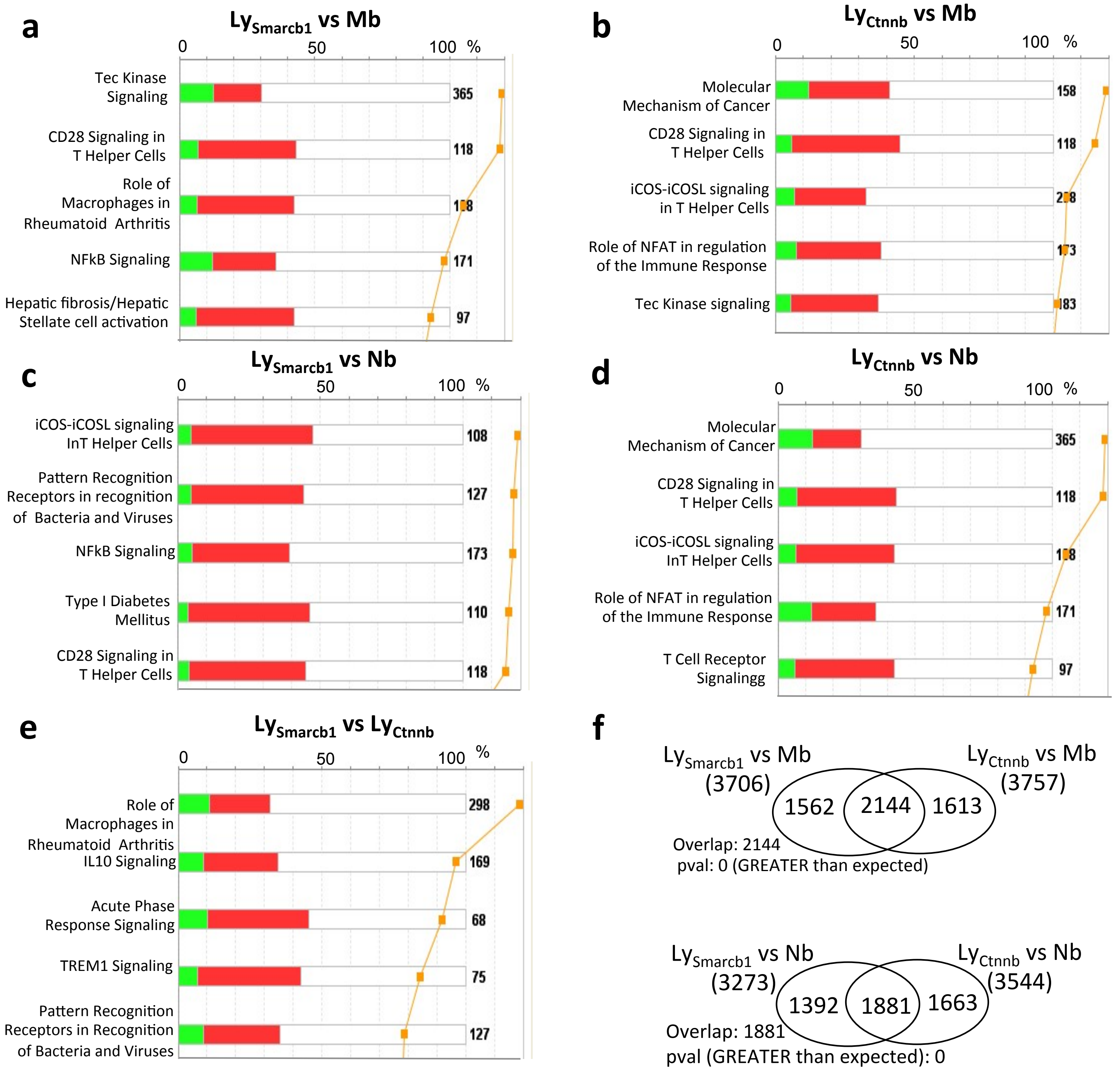

Ingenuity analyses of genes with significantly different expression (t-test analyses, p≤0.05, Fold Change |FC|≥1.2) in *Smarcb1*-deficient mouse lymphomas ($Lymph_{Smarcb1}$) as compared with Mb (a) and Nb (c); similar analyses comparing *Ctnnb*$^{del\_ex3}$ T-cell lymphomas ($Lymp_{Ctnnb1}$) with Mb (b) and Nb (d); similar analysis between the two types of lymphomas (e). Pathways are ranked from the most (up) to the less (down) significantly represented ones. The columns' length represents the percentage of genes in the pathway that are significantly differentially expressed. Genes involved in the pathways are numbered at the end of the columns. Genes overexpressed in lymphomas as compared with Mb are shown in red, under-expressed genes in green. The yellow curve indicates the p.value for each overrepresented pathway. (f) Significant overlaps of the differential expressions between each type of lymphomas and Mb (upper panel) and Nb (lower panels).

Supplementary Figure 2. Rosa26-Cre dependent recombination throughout mouse development

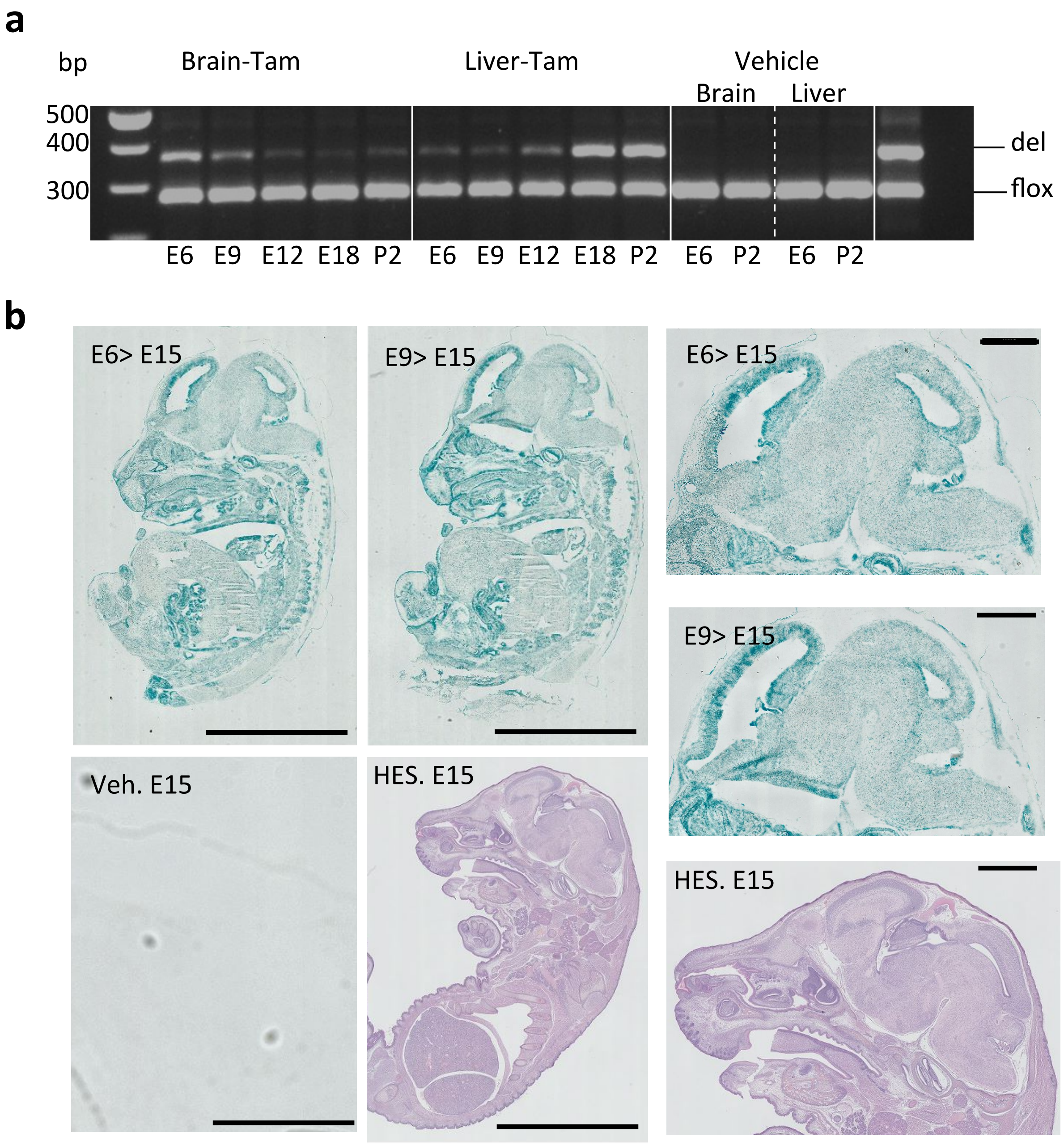

(a) Semi-quantitative PCR on mouse brains and livers, five days after treatment with tamoxifen (Tam, 1mg/20g) and vehicle at various time points (from E6 to birth). Del: deleted allele; flox: floxed allele. (b) Similar diffuse β-galactosidase staining in brains at E15 in Rosa26-Cre^{ERT2};Rosa26-LacZ mice treated with tamoxifen at E6 (left upper panel) and E9 (right upper panel) ; scale bars represent 5mm. HES staining of control mouse at E15. Right panel: higher magnification focusing on brains ; scale bars for high magnification represent 1mm

Supplementary Figure 3. Tamoxifen injection at various stages of development

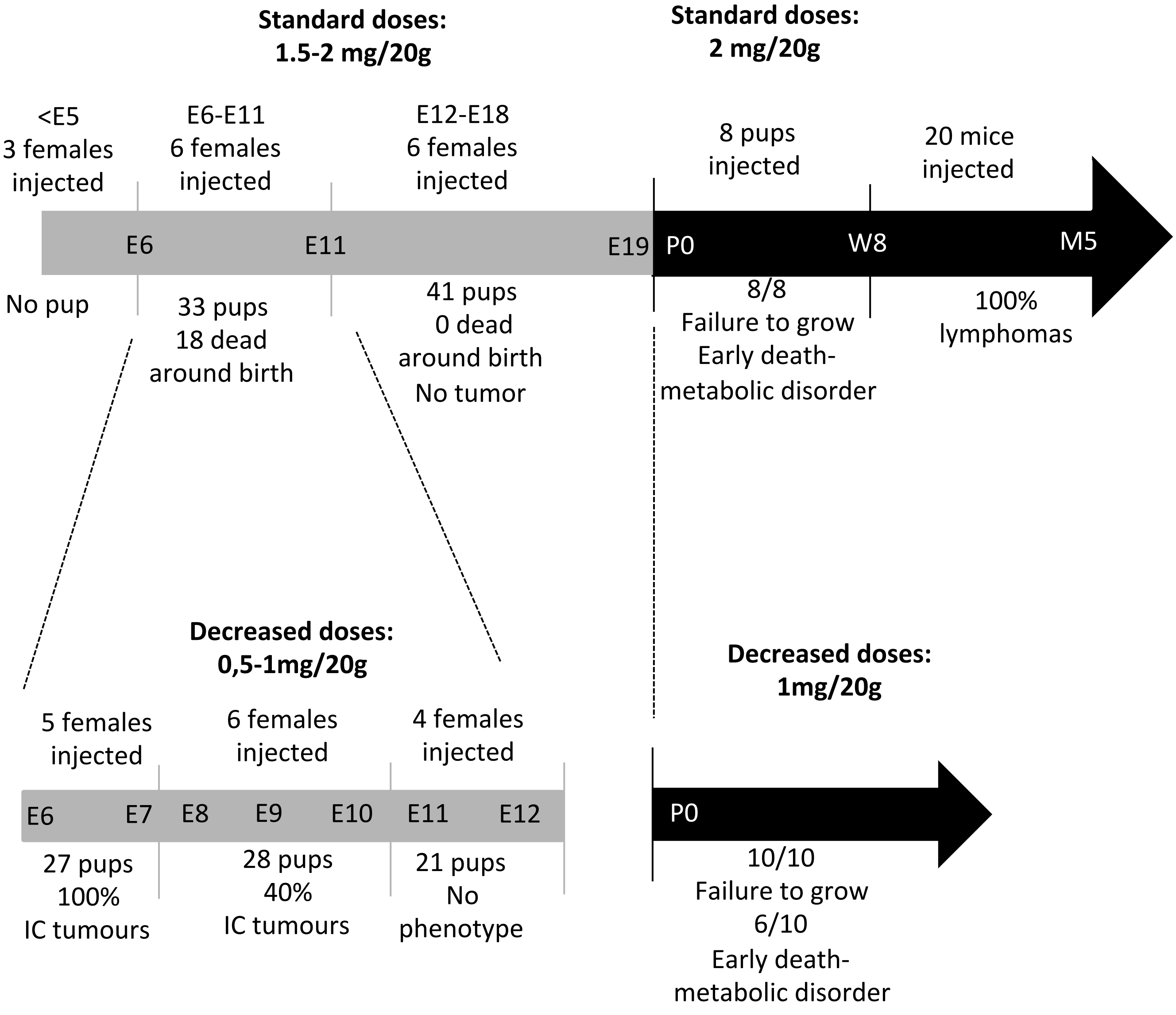

P0: birth day. W8: 8 weeks of life. M5: 5 months of life. Mice showed no phenotype when treated with tamoxifen from E12 to E18 with standard doses (1.5-2mg/20g); therefore, we didn't use reduced doses after E12.

Supplementary Figure 4. Mouse intra-cranial tumors are split in two entities

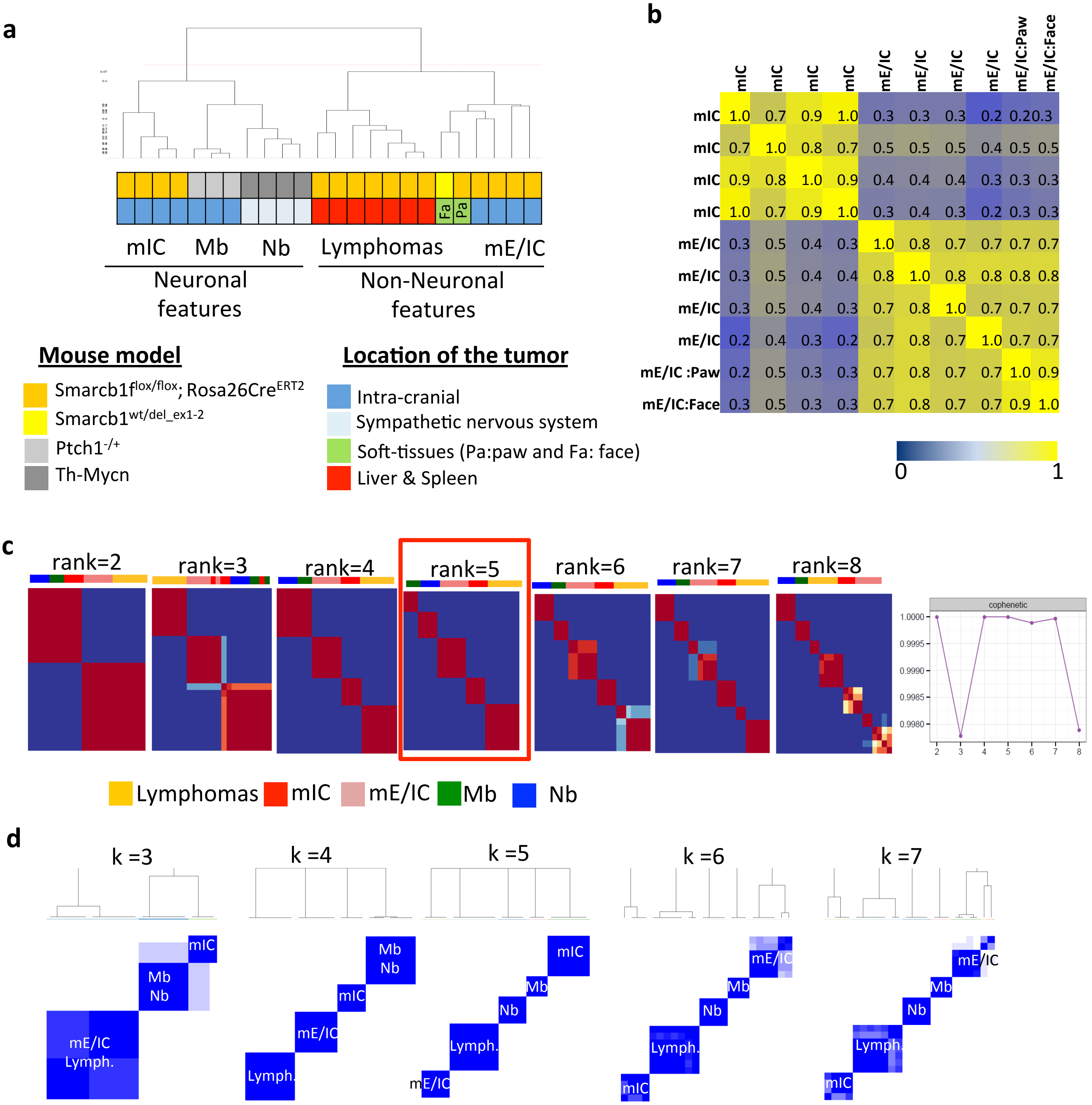

(a) Unsupervised hierarchical clustering on 3161 genes; mIC clusterize with neuronal tumors (Mb and Nb) while mE/IC clusterize with lymphomas (b) whole genome correlation between *Smarcb1*-deficient mouse tumors; a high correlation is found between intracranial tumors of the mE/IC group and the soft-tissue tumors. i.e. mE/IC arising in the paw (pa) and the face (fa). (c) Non Negative Matrix Factorization on the whole set of mouse *Smarcb1*-deficient tumors . Mb and Nb; the most robust and lowest number of clusters that split Mb from Nb is k=5: Mb, Nb, the Lymphoma group and 2 distinct groups within *Smarcb1*$^{flox/flox}$;Rosa26-Cre^{ERT2} intracranial mouse tumors. (d) Consensus clustering on the same set of tumors showing that a number of clusters lower than 5 is unable to split Mb from Nb.

Supplementary Figure 5. Human intra-cranial tumors are split in three entities

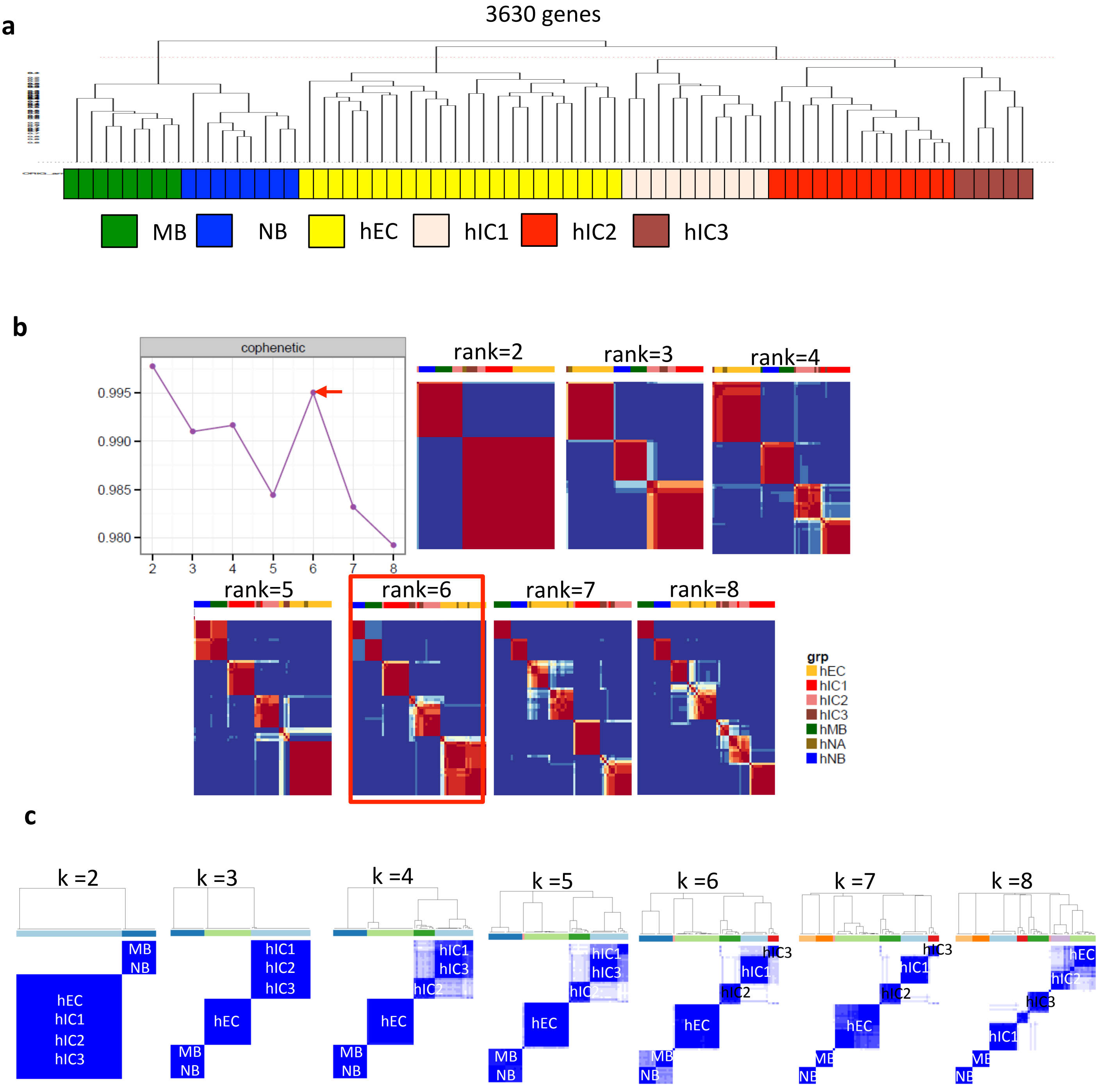

(a) Unsupervised hierarchical clustering on 3630 genes; 4 clusters within all *SMARCB1*-deficient tumors; hEC: human extra-cranial tumors; hIC: human extra-cranial tumors, groups 1, 2 and 3: MB: human medulloblastomas; NB: human neuroblastomas; NA: non assigned (b) Non Negative Matrix Factorization on the whole set of human *SMARCB1*-deficient tumors, MB and NB; the most robust (highest cophenetic coefficient) and lowest number of clusters that split MB from NB is 6, confirming 4 distinct groups within *SMARCB1*-deficient tumors. (c) Consensus clustering on the same set of tumors, showing that a number of cluster lower than 6 is unable to split MB from NB.

Supplementary Figure 6. RT-PCR confirmation on a subset of selected genes

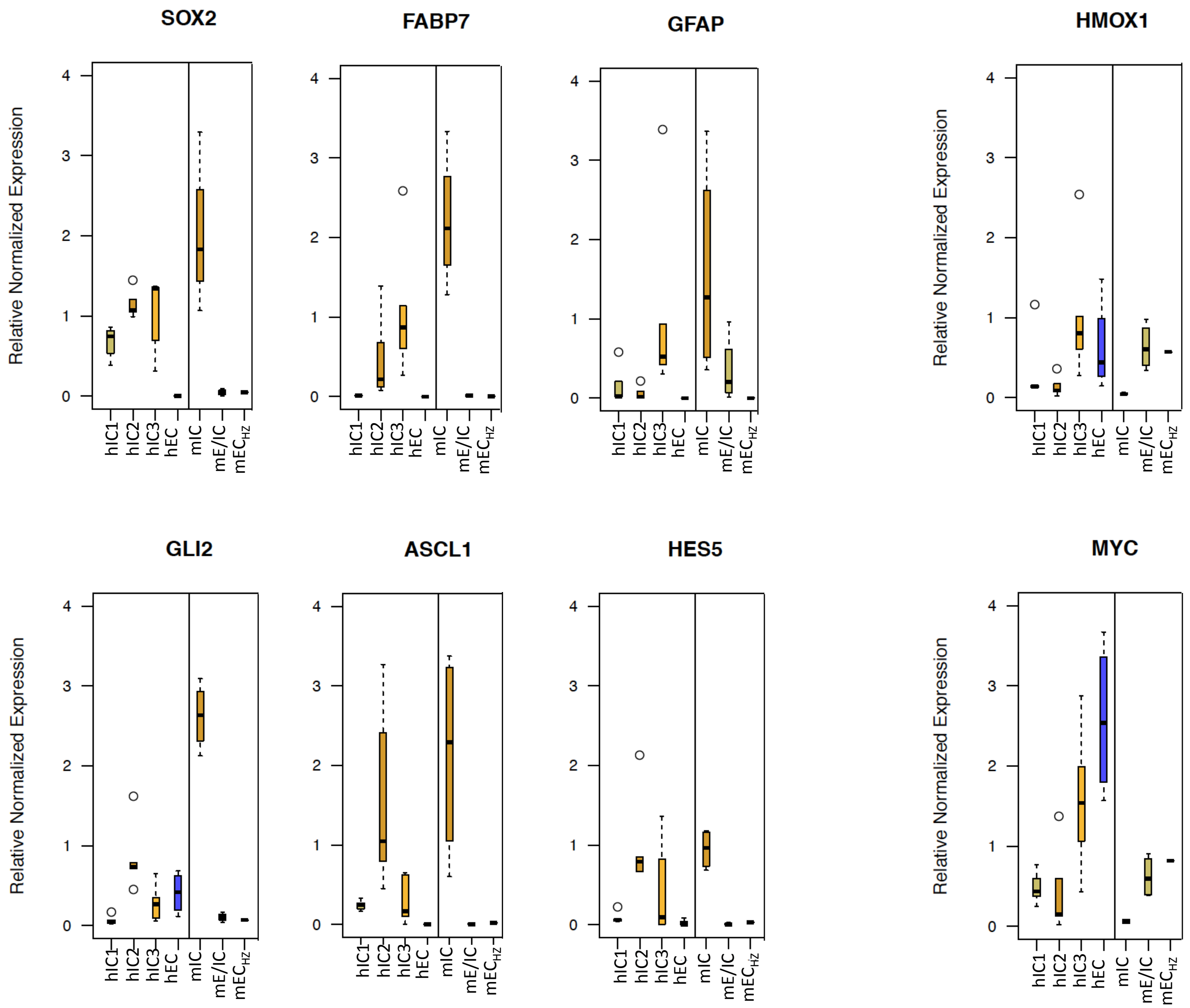

8 genes that distinguish mE/IC from mIC were assessed on human tumor subgroups (hEC, n=5 ; hIC1, n=5, hIC2, n=5, and hIC3, n=5) and mouse tumors subgroups (mIC, n=4, and mE/IC, n=4); the levels of expression in the face tumor from the heterozygous model (mEC$_{Hz,}$ n=1) are also separately depicted to show the similarity of their expression profile with other tumors from the mE/IC group. In box-plots, the central rectangle spans the first quartile to the third quartile (*interquartile range* or *IQR*); the horizontal line inside the rectangle shows the median; whiskers are taken to 1.5×*IQR* from the quartile ; circles show outliers

Supplementary Figure 7. *ACTL6A* and *ACTL6B* RNA expression

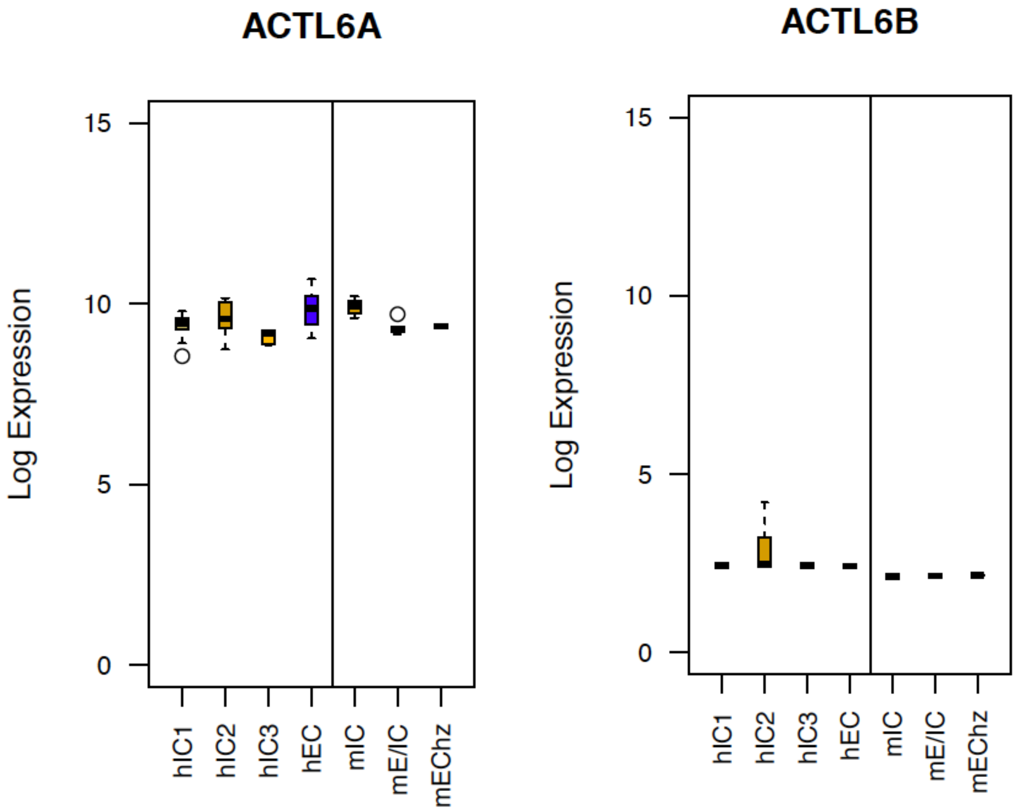

In box-plots, the central rectangle spans the first quartile to the third quartile (*interquartile range* or *IQR*); the horizontal line inside the rectangle shows the median; whiskers are taken to 1.5×*IQR* from the quartile ; circles show outliers hIC1, n=11 ; hIC2, n=;12 hIC3, n=5; hEC, n=20; mIC, n=5; mE/IC, n=4; mEC_{hz}, n=1.

Supplementary Figure 8. Full gel showing the PCR results on wild type, knock out (KO) and floxed alleles of *Smarcb1*

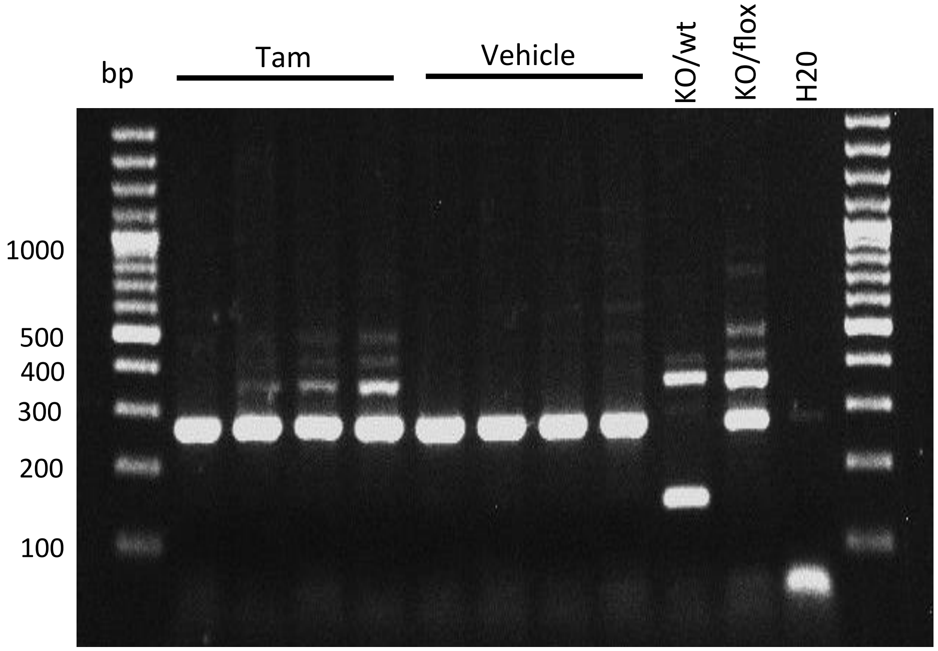

bp: base pair; Tam: tamoxifen; wt: wild type.

Supplementary Table 1. Existing mouse models restrictedly based on *Smarcb1* inactivation

	Genetic background	Tumour penetrance	Phenotype	Mean Latency
Guidi et al, Moll Cell Biol, 2001	Promoter trap in intron 3	15%	Head and neck soft-tissues tumours Sarcomas, lymphoma	25weeks
Klochendler-Yeivin et al, EMBO, 2000	Deletion of exons 1 & 2	32%	Intracranial paraspinal subcutaneous tumors Pleomorphic Rhabdoid cells	9 months
Roberts et al, PNAS, 2000	Deletion of exon1	6.5%	Face and Neck soft-tissue tumors Pleomorphic Rhabdoid cells	6 months
Tsitikis et al, PNAS, 2005	Deletion of exons 6&7	26%	Face and neck Soft-tissue tumors Pleomorphic Rhabdoid cells	11 months
Roberts et al, Cancer Cell, 2002	Conditionnal Inversion	100%	Spleen and liver lymphomas	3months
Moreno et al, J Neuroscience, 2015	Promoter specific for granule cell precursors : Atoh1-Cre	0%	Cerebellum hypoplasia	Not applicable

Supplementary Table 2. Primers used for RT-PCR validation of gene expression

	Sense	Anti-sense
**Human SOX2**	GCCCCCAGCAGACTTCACAT	AGGGGCAGTGTGCCGTTAAT
**Human FABP7**	TCATCAGGACTCTCAGCACATTCAA	CCATCCAGGCTAACAACAGACTTACA
**Human GAFP**	CAGAAGCTCCAGGATGAAACCAA	GTGGCTTCATCTGCTTCCTGTCT
**Human ASCL1**	CGTCCTGTCGCCCACCATCT	GGGGCTGAGCGGGTCGTAA
**Human HES5**	GAAGCACAGCAAAGCCTTCGT	GTAGCCTTCGCTGTAGTCCTGGT
**Human GLI2**	AAGTCACTCAAGGATTCCTGCTCA	GTTTTCCAGGATGGAGCCACTT
**Human MYC**	ACCACCAGCAGCGACTCTGA	TCCAGCAGAAGGTGATCCAGACT
**Human HMOX1**	CAGTCAGGCAGAGGGTGATAGAAGA	CTGCAACTCCTCAAAGAGCTGGAT
**Mouse SOX2**	CACATGGCCCAGCACTAC	CCCTCCCAATTCCCTTGTATC
**Mouse FABP7**	TGTAAGTCTGTGGTTCGGTTG	AGGGGCAGTGTGCCGTTAAT
**Human FABP7**	TCATCAGGACTCTCAGCACATTCAA	AGCAACGATATCCCCAAAGG
**Mouse GAFP**	GAAAACCGCATCACCATTCC	CTTAATGACCTCACCATCCCG
**Mouse ASCL1**	GACTTGAACTCTATGGCGGG	TTCCAAAGTCCATTCCCAGG
**Mouse HES5**	CGGTGGAGATGCTCAGTC	CTTGGAGTTGGGCTGGTG
**Mouse GLI2**	GCTCCACACACCCGCAACA	AAGTTTTCCAGGACAGAACCATTGA
**Mouse MYC**	ACCACCAGCAGCGACTCTGA	GGAATGGAGATGAGCCCGACT
**Mouse HMOX1**	ACAGAGGAACACAAAGACCAG	GTGTCTGGGATGAGCTAGTG
